# Impact of COVID-19 on migrants’ access to primary care and implications for vaccine roll-out: a national qualitative study

**DOI:** 10.3399/BJGP.2021.0028

**Published:** 2021-06-15

**Authors:** Felicity Knights, Jessica Carter, Anna Deal, Alison F Crawshaw, Sally E Hayward, Lucinda Jones, Sally Hargreaves

**Affiliations:** The Migrant Health Research Group, St George’s, University of London, London.; St George’s, University of London, London.; The Migrant Health Research Group, St George’s, University of London, London.; The Migrant Health Research Group, St George’s, University of London, London.; The Migrant Health Research Group, St George’s, University of London, London.; Palladium, London.; The Migrant Health Research Group, St George’s, University of London, London.

**Keywords:** COVID-19 vaccines, primary health care, delivery of health care

## Abstract

**Background:**

COVID-19 has led to big changes in UK primary care, including rapid digitalisation, with unknown impact on migrant groups.

**Aim:**

To understand the pandemic’s impact on recently-arrived migrants and their access to primary health care, and implications for vaccine roll-out.

**Design and setting:**

Qualitative study involving semi-structured interviews with primary care professionals (PCPs) and migrants in urban, suburban, and rural settings across England.

**Method:**

Sixty-four PCPs and administrative staff, and 17 recently-arrived migrants were recruited using purposive, convenience, and snowball sampling. In-depth, semi-structured interviews were conducted by telephone. Data were analysed iteratively, informed by thematic analysis.

**Results:**

PCPs and migrants concurred that digitalisation and virtual consultations have amplified existing inequalities in access to health care for many migrants, due to a lack of digital literacy and access to technology, compounded by language barriers. PCPs were concerned that virtual consultations resulted in difficulties building trust and risked missing safeguarding cues. Both PCPs and migrants highlighted challenges around registering and accessing health care due to physical closure of surgeries, as well as indirect discrimination, language and communication barriers, and a lack of access to targeted and tailored COVID-19 information or interventions. Migrants reported a range of specific beliefs, from acceptance to mistrust, around COVID-19 and potential COVID-19 vaccines, often influenced by misinformation. Innovative opportunities were suggested, including translated digital health advice using text templates and YouTube; these merit further exploration.

**Conclusion:**

Pandemic-related changes to primary care delivery may become permanent; some migrant groups are at risk of digital exclusion and may need targeted additional support to access services. Solutions are needed to address vaccine hesitancy in marginalised groups to ensure equitable COVID-19 vaccine uptake.

## INTRODUCTION

Migrants to the UK — particularly recent arrivals and marginalised groups, including refugees and asylum seekers — may face barriers to accessing primary care.^1^ These include confusion about the NHS, language difficulties and discrimination, and, for some, restricted entitlement to health care due to their immigration status.^2^ Migrants are a diverse group, but are considered to be disproportionately impacted by infectious diseases compared with the host population and may be underimmunised, with implications for health systems on arrival.^3^ There are concerns that the COVID-19 pandemic has exacerbated these inequalities; emerging data highlights the differential impact of SARS-CoV-2 on migrant groups and the wider population of minoritised ethnic groups,^4^^–^^6^ along with limited exploration to date of the views of migrants themselves.

The digitalisation of primary care has been a key feature of the pandemic, with countries moving from face-to-face to virtual consultations — that take place via telephone, video, and online form-based communications (including eConsult) — and text communications (including accuRx).^7^^,^^8^ A longitudinal mixed-methods study on the implementation of remote consulting in UK general practice demonstrated a rapid change: 90% of GP appointments and 46% for nurse appointments were held remotely in April 2020 in the 21 practices studied.^9^ Text messaging increased more than threefold in April to July 2020 compared to 2019.^9^ Digitalisation, however, can disproportionately disadvantage marginalised groups, amplifying existing structural inequalities through differential access and digital literacy, differential capacity to benefit, and differential motivation for use.^10^^,^^11^ A recent report on migrants living in vulnerable circumstances in England described an inability to register with a GP, digital exclusion, and language barriers as preventing access to health advice for those with COVID-19 symptoms.^12^ These barriers may have important implications for the COVID-19 vaccine roll-out, with Public Health England suggesting flexible delivery models to reduce inequalities in vaccine uptake in at-risk populations from Black, Asian, and minority ethnic groups,^13^ which includes some migrant populations.

This study aimed to seek the views of both a range of primary care professionals and recently-arrived migrants to the UK (residing in the UK for <10 years) to:

- explore the specific impact of the pandemic on migrants and their access to primary care;
- determine the implications for COVID-19 vaccination uptake; and
- better understand potential solutions to inform the immediate public-health response.

**Table:** How this fits in

This study revealed that the rapid digitalisation of primary care services and physical closure of surgeries during the pandemic have amplified disparities in access to health care for specific migrant groups, with many lacking access to, and the capacity to use, technology; this is compounded by language barriers. Migrants may be at increased risk of misinformation about COVID-19 and face barriers to vaccination, which merits further consideration as the COVID-19 vaccine roll-out continues. Improved outreach to local migrant community organisations and places of worship, alongside codesigning with migrants more-inclusive delivery approaches and creative integration of migrant ambassadors into information-sharing campaigns, are needed. Primary care can maximise the opportunities of digitalisation through engagement by multiple modalities (for example, text, email, letter, and YouTube videos) to provide targeted, translated advice, virtual group consultations, and work with local leaders to access and disseminate information through informal communication channels.

## METHOD

### Study design

An in-depth qualitative study of recently-arrived migrants and primary care professionals was undertaken. This semi-structured interview study involved three iterative, linked phases, with each phase informing data collection and analysis for the subsequent phase:

- phase one — interviews with clinical primary care professionals;
- phase two — interviews with administrative primary care professionals; and
- phase three — interviews with recently-arrived migrants (born overseas, aged >18 years, and <10 years in the UK).

Topic guides were developed by the research team, which comprised two GPs and four academic researchers; they were then piloted by the two GPs, with input from the team’s migrant representative project board. Several migrant representatives offered ongoing feedback throughout the study. Separate topic guides were developed for each phase.

Primary care professionals were asked about their experience of providing health care to migrants, the impact of the COVID-19 pandemic, and implications for the COVID-19 vaccine roll-out. Views were sought from migrants about their experiences during the pandemic, the impact on access to primary health care, and their views on the COVID- 19 vaccine delivery in their communities. Further details on the topics and questions are outlined in Table 1.

**Table 1.** Foci of topic guides: summary

**Focus**	**Aim**	**Example question(s) for primary care professional**	**Example question(s) for migrant**
**Orientation**	To elicit the lived experience and context from which the participant was speaking about core topics in the interview	Do you regularly see migrant patients as part of your daily practice? Have you had any training in relation to migrant health?	Can you tell me about your migration status? Do you have a permanent and full registration with a GP in the UK?
**Pre-pandemic barriers**	To provide a comparison to the participant’s perspective about what has changed as a result of the pandemic	What is your practice’s approach to migrant patients? Why do you think this group is hard to reach?	How long after arriving in the UK did you first contact a GP or any other healthcare service, and what were your experiences/thoughts on this process?
**General impact of the pandemic**	To understand the perceived context of the pandemic on the specific topics of interest	Has COVID-19 altered the way your practice functions?	How has the pandemic impacted the migrant community around you?
**Impact of the pandemic on access to primary care**	To explore how the participant’s perceptions of migrants’ access to primary care has been affected, for what reasons, and any potential solutions to barriers identified	What have been the challenges and opportunities of COVID-19 for the way your practice functions for migrant patients?	How do you think COVID-19 affected your ability to seek health care during the pandemic?
**COVID-19 vaccine uptake in migrants**	To explore participants’ perceptions on vaccine hesitancy in migrant groups, the underlying reasons, and potential solutions to this	If COVID-19 vaccine is targeted at adults, do you think there will be any issues regarding uptake in migrant populations at your practice?	Are there any groups that may be more unlikely/less willing to be vaccinated with the COVID-19 vaccine? Which ones? Why? Can you think of any barriers/facilitators that are specific to this vaccine for other migrants around you?

### Sampling and recruitment

In phases one and two, primary care professionals were recruited using purposive sampling in urban, suburban, and rural settings across England. Further participants were identified by means of ‘snowballing’ — namely, those recruited were asked to contact colleagues. Recruitment packs were disseminated through six local clinical research networks (CRNs) — CRN Kent, Surrey and Sussex; CRN South London; CRN North Thames; CRN North West London; CRN West Midlands; and CRN Greater Manchester — and advertised in GP bulletins, newsletters, and through practice manager mailing lists. Additional participants were recruited from Newcastle and Oxford through word of mouth.

In phase three, migrants were recruited using convenience and snowball sampling. Advertisements for the study and Englishlanguage participant information sheets were circulated across England to 20 migrant support groups and charities providing healthcare-related support to migrants, and on social media. Further participants, including those who did not speak English, were recruited by word of mouth. Verbal explanation over the telephone with an interpreter was offered to interested individuals; interpreters were available for interviews as required.

### Research team and reflexivity

The team comprised two GPs and four academics, with specific input from a wider project board comprising migrant representatives from a range of different nationalities and backgrounds. This diversity enabled robust discussion throughout the design, collection, and analysis stages, and the team attempted active reflexivity throughout. The two GPs attempted to overlook their profession before, during, and after the interviews with primary care professionals.

### Ethical approval and informed consent

Ethical approval was granted and for all three phases participant information sheets were circulated; signed, informed consent was received prior to telephone interview and participants consented to their interviews being audiorecorded.

### Data collection and analysis

In-depth, semi-structured interviews lasting 30–90 minutes were conducted by telephone by three academic researchers and the two aforementioned GPs. Two interviews with migrants were conducted with interpreters. Semi-structured interviews were used as they not only enable targeted questioning but also provide the opportunity for participants to explore, in detail, the areas of importance to them.

Participants were compensated with shopping vouchers (primary care professionals received £20; migrants received £37 because their interviews took longer). Each interview was audiorecorded, transcribed verbatim, checked for accuracy, and anonymised. Data collection ended when data saturation was reached in key themes across participant groups as described by Saunders *et al,*^14^ and when new data demonstrated redundancy to existing data in line with the approach recommended by Grady;^15^ data were analysed inductively, informed by thematic analysis. This allows for the iterative analysis of collective experiences across a dataset.^16^ The two GPs undertook immersion as advocated by Ritchie and Spencer.^17^ Transcripts were analysed using NVivo (version 12). A comprehensive code list was developed by one GP, then agreed by the other GP and two of the academic researchers; disagreements were resolved through negotiated consensus and key themes were conceptualised through further discussion with the wider team.

## RESULTS

In total, 81 interviews were conducted between 18 June and 30 November 2020. In phase one, 48 interviews were held with primary care professionals, — namely, 25 GPs, 15 practice nurses (PNs), seven healthcare assistants (HCAs), and one clinical pharmacist. In phase two, interviews were held with 16 administrative staff (11 practice managers and five receptionists/other). Participant characteristics are outlined in Table 2 (mean age 45 years; 84.4% female; and a range of ethnicities).

During phase three, 17 interviews were conducted with migrants: 15 (88.2%) with asylum seekers, and two with refugees (64.7% female; mean age 38 years [range: 22–59 years]; mean time in the UK 4 years [range: 9 months–9 years]). Participants originated from 14 countries across five World Health Organization regions (Table 3).

**Table 2.** Participant characteristics: primary care professionals

**Characteristic**	**All participants**	**GP**	**Practice nurse**	**Practice manager/administration team**	**Healthcare assistant/clinical pharmacist**
***n* (%)**	64 (100.0)	25 (39.1)	15 (23.4)	16 (25.0)	8 (12.5)

**Age, years, mean (SD)**	45 (11.8)	44 (11.0)	45 (11.9)	48 (11.6)	41 (12.7)

**Sex, *n* (%)^a^**					
Female	54 (84.4)	17 (26.6)	15 (23.4)	14 (21.9)	8 (12.5)
Male	10 (15.6)	8 (12.5)	0 (0.0)	2 (3.1)	0 (0.0)

**Ethnicity, *n* (%)**					
African	4 (6.3)	0 (0.0)	3 (4.7)	0 (0.0)	1 (1.6)
Other Asian background	2 (3.1)	1 (1.6)	1 (1.6)	0 (0.0)	0 (0.0)
Mixed	3 (4.7)	1 (1.6)	0 (0.0)	1 (1.6)	1 (1.6)
Other White	5 (7.8)	1 (1.6)	2 (3.1)	1 (1.6)	1 (1.6)
Caribbean	1 (1.6)	0 (0.0)	0 (0.0)	0 (0.0)	1 (1.6)
Indian	11 (17.2)	8 (12.5)	0 (0.0)	3 (4.7)	0 (0.0)
Pakistani	3 (4.7)	2 (3.1)	0 (0.0)	1 (1.6)	0 (0.0)
White British	32 (50.0)	12 (18.8)	9 (14.1)	9 (14.1)	2 (3.1)
White Irish	3 (4.7)	0 (0.0)	0 (0.0)	1 (1.6)	2 (3.1)

**Practice size, *n* (%)**					
<5000	6 (9.4)	1 (1.6)	1 (1.6)	2 (3.1)	2 (3.1)
5000–10 000	24 (37.5)	10 (15.6)	2 (3.1)	9 (14.1)	3 (4.7)
10 000–15 000	10 (15.6)	6 (9.4)	2 (3.1)	0 (0.0)	2 (3.1)
15 000–20 000	15 (23.4)	6 (9.4)	4 (6.3)	4 (6.3)	1 (1.6)
>20 000	9 (14.1)	2 (3.1)	6 (9.4)	1 (1.6)	0 (0.0)

**Practice location, *n* (%)**					
Rural	1 (1.6)	1 (1.6)	0 (0.0)	0 (0.0)	0 (0.0)
Suburban	13 (20.3)	7 (10.9)	2 (3.1)	2 (3.1)	2 (3.1)
Urban	50 (78.1)	17 (26.6)	13 (20.3)	14 (21.9)	6 (9.4)

a *Percentages are calculated from the total study cohort. SD* = *standard deviation.*

**Table 3.** Participant characteristics: migrants

**Characteristic**	***n* (%)^a^**
**Age, years^b^**	
Mean (SD)	38 (8.4)
<35	5 (33.3)
35–50	9 (60)
>50	1 (6.7)

**Sex**	
Female	11 (64.7)
Male	6 (35.3)

**WHO region of origin**	
African (Mauritius, Nigeria, Zimbabwe, and other unstated)	4 (23.5)
The Americas (Venezuela)	1 (5.9)
Eastern Mediterranean (Afghanistan, Egypt, Iraq, Pakistan, and Palestine)	5 (29.4)
European (Kyrgyzstan, Turkey, and Ukraine)	3 (17.6)
South East Asian (Sri Lanka)	4 (23.5)

**Time since arrival in the UK, years, mean (SD)**	
<2	2 (11.8)
2–5	9 (52.9)
6–10	6 (35.3)

a *Unless otherwise stated.*

b *Missing data,* n= *2. SD* = *standard deviation. WHO* = *World Health Organization.*

### Impacts of the COVID-19 pandemic on migrants’ access to primary care

There was marked convergence on themes between participants; they reported multiple pandemic-related impacts, risk factors for contracting SARS-CoV-2, and issues regarding the COVID-19 vaccine roll- out, as well as a range of opportunities to improve access to primary care.

#### Digitalisation

Primary care professionals described a shift to digitalisation of registration, appointments, and the giving of health information and prescriptions by text; they largely agreed that this was ‘here to stay’. Although some primary care professionals disagreed, many were concerned that a lack of technology, along with challenges in using it, are barriers to access:

*‘One of the things that’s being pushed forward is remote consulting through eConsult, for example … If you build more roads, you increase the traffic. So you’re not actually dealing with the demand in people who actually need the care. And a lot of migrant populations are absolutely fine with technology. But, again, the outreach of that technology, or how to access it, isn’t known to them. And I feel the technology thing* [inequality of access] *in the pandemic is going to widen.’*

(GP8)

Some primary care professionals reported reduced migrant registrations or attendances compared to pre-pandemic, which were speculated to arise either due to the challenges migrants experienced with digitalisation or due to increased fear of COVID-19 and a preference for home remedies:

*‘Migrant patients did not want to come in. They tended to stay in the household … maybe they feel that at home, they’re safe … they do home remedies.’*

(HCA6)

Others perceived that digitalisation had increased access for patients who were young, fit, and non-migrants, while exacerbating the exclusion of marginalised patients:

*‘Because they are easily put off … You know it’s perfectly fine for me as a young, white, middleclass woman from here. I’ll call my GP and make a fuss till I’m seen.* [But for migrant patients] *there’s a barrier on the phone and the barrier of expectation ... they are easier to ignore and it’s quite an issue with the technology, so that’s another marginalisation inequity* [for migrant patients]. *’*

(GP24)

Digitalisation affected many migrants; responders cited a lack of ownership of technology or not knowing how to use it, or being unable to afford to maintain it:

*‘They ask you to go onto the website, fill out the form, sign it, scan it, and then send it back to them, so they can register you. I mean, I don’t have a scanner, I don’t have printers, then how can I kind of download it, scan? Or, if I can do it online, like an electronic signature, most people don’t know how to apply that. You need a computer. You can’t do that on your phone. So, those forms, for example, are not accessible at all for many people.’*

(Migrant 9)

Other primary care professionals, particularly practice managers, reported that technology has presented actual and potential solutions for migrant groups, such as translating texts into the patient’s language, targeted digital communications to encourage access, group video consultations, and YouTube videos to deliver health advice:

*‘I’ve been texting in Turkish. I answer all my Turkish patients and I just know enough Turkish to check I’m not saying complete gobbledegook with Google Translate.’*

(GP16)

*‘We do an awful lot of stuff by text as we find that works really well and across language barriers … Migrant people really locked onto* [YouTube] *because you can see. It just works.’*

(Administration team member 8)

*‘We sent out some text messages, just saying, “we’re here, we are open, please come and see us … ”. One of our receptionists is making phone calls to where we had very vulnerable migrant families. We also made some COVID leaflets out as well, that was in Somali language and different languages.’*

(HCA6)

#### Social and economic factors

Concerns were cited by both primary care professionals and migrants that migrants risked pandemic-related financial insecurity, and may have faced increased exposure to SARS-CoV-2 due to front-facing jobs:

*‘Because of COVID, there’s going to be a lot more job insecurity for these people, which is going to have more of an impact on their health care anyway. So I think we’re going to see a lot of problems in terms of poverty and food banks and people who’ve got no recourse to public funds.’*

(PN13)

Migrants often expressed that financial concerns, social exclusion, and poor living conditions resulting from the pandemic had a substantial negative impact on mental health:

*‘It’s* [the pandemic and lockdown] *just making the situation for people worse, in a way, because people will start having suicidal thoughts, starting to think about the country that you came here from, there’s war, there’s poverty.’*

(Migrant 14)

However, some primary care professionals reported increased support for access for marginalised groups during the pandemic:

*‘I think migrants are more likely to be destitute, and we give out food bank vouchers and free-food lists, but a lot of our work ... Certainly not so much during COVID because of the policy of putting people in hotels* [including provision of regular meals] *etc ... Much of my work before COVID was trying to get people somewhere to live and get them funds and food, and I think that’s a huge part of work with ... not just migrants in that situation, but more likely to be migrants.’*

(GP7)

Migrant participants highlighted being moved into cramped hotel or hostel accommodation, and raised concerns around additional costs resulting from the pandemic — for example, due to needing to buy soap and masks when they are on very low budgets:

*‘We live on £5, £6 daily so, on top of this, you have to buy soap and you have to buy disinfectant, you have to buy a mask — it adds a lot of pressure on your budget.’*

(Migrant 17)

They also reported a loss of access to support networks and community organisations during the pandemic, services that previously helped them to access health care and navigate the healthcare system:

*‘Before the pandemic, you know, people who are British volunteers used to help us, speak to the refugees to apply for* [help with healthcare costs] *. But now everything is closed.’*

(Migrant 5)

Clinical primary care professionals were concerned about the increased risk factors in migrants making them vulnerable to contracting, and suffering serious illness from, COVID-19:

*‘The* [migrant] *population is a very high-risk population, because of obesity and diabetes, ethnicity and other comorbidities … we have seen a lot of people dying.’*

(GP22)

#### Language barriers

Language barriers were repeatedly reported by migrants and primary care professionals alike, and were perceived to have increased due to digitalisation (for example, closed surgeries necessitated a reliance on virtual consultations, and online forms being in English only):

*‘I think a face-to-face consultation between a recently arrived migrant, particularly the language barrier, is really, really difficult. And I think the phone conversations that I’ve had* [because of the pandemic] *have been significantly more so, to the point that, if there’s going to be a language barrier, and I think it’s a complex problem, I’ll just book people in for a face-to-face* [consultation] *.‘*

(GP15)

Some migrants reported that lockdowns had reduced access to friends who had previously translated for them, and had had a negative impact on their ability to understand health information, appointment letters, and messaging around COVID-19:

*‘None of them speaks English so they were not aware about the restriction and what is the rules. So I advised them: wearing face masks, washing their hands … if I don’t speak English and I get a letter, I could travel to go seek help from my friends* [who could] *translate for me. But what if I don’t speak English and there is a lockdown where I cannot go out? ’*

(Migrant 4)

Some GPs expressed a lack of knowledge or desire to engage with virtual consultations involving an interpreter, while several GPs and PNs highlighted concerns about confidentiality and their ability to detect cues and safeguarding concerns virtually:

*‘I think if there are language barriers, then absolutely* [telephone consultations cause challenges for migrants] … *I imagine that must be quite challenging because you’ve got to sort out how to do that* [interpreted consultation] *three-way.’*

(GP3)

*‘I mean confidentiality is another issue in terms of people’s living situation and overcrowding, and maybe they’re sharing computers or obviously rooms and phone calls. We don’t know who’s in the background when we ring people … there are quite a lot of safeguarding issues.‘*

(GP18)

However, some primary care professionals reported an improved ability to organise language support, and improved access through digital consultations:

*‘The e-consultation method has, surprisingly, shown how the migrant contacts with the surgery have actually increased, compared to pre-COVID … And increased the reach towards patients who might have language barriers, because they have the ability now to take their time — maybe use a translator when they’re writing, and write down their concerns.’*

(GP1)

#### Trust, authority, and information

Both migrants and clinical primary care professionals, particularly HCAs, commented on a lack of information targeted at migrants about access to health care, public-health messages about COVID- 19, and the COVID-19 vaccine itself:

*‘I think the biggest problem* [for COVID-19 vaccine uptake] *is going to be language and culture … It’s been very blustery from politicians. If English wasn’t your* [first] *language and you watched a press conference, it’s quite hard to work out what is going on actually. And then the public-health messaging: again, it’s not always been very simplistic. It has been changing. It’s only because organisations like Doctors of the World* [providing translated resources] *… The big issue, basically, is language getting out there to people who need it. The people that need it, it probably won’t get to them because they’re not interacting with their health necessarily in the way that the healthcare system was built to do, if that makes sense. The healthcare system is mainly built for fairly tech-literate, Englishliterate people. And they’re not always using the same channels.’*

(GP8)

Migrants reported not understanding health-service changes, considerable misinformation circulating among migrant communities about COVID-19, and the COVID-19 vaccine:

*‘Some of the people, culturally, they don’t believe that such a virus exists. They think that it’s 5G or something else. They rely on other news, so, that’s why, in order to change their minds and kind of make them believe, there should be an effective system of information.’*

(Migrant 6)

*‘And we had several issues which were urgent — for example, my husband, he couldn’t move, he had a pain in his back. But we felt we can’t go to the GP because of this COVID. I worried. I thought, what if it’s very, very urgent, what do we do? Even now, I don’t know. If something’s urgent, is the emergency department working at the moment?’*

(Migrant 15)

Both migrants and primary care professionals stated that some migrant patients have low levels of health literacy and do not believe in, or trust, science, the UK health system, or government, and tend to seek religious or peer input into decision making:

*‘* [Social-media groups] *were spreading a lot of information like “don’t go outside tonight because the government will be spreading the powder that will stop COVID”. And the funny thing is people believe it because somebody sent them* [the information] *… Like I see in the Russian-speaking group on Facebook so much confusion, so much misunderstanding of the system … I think this is where people make decisions. They will not trust a GP. Even after 16 years in the country.’*

(Migrant 8)

*‘I think they follow advice, and healthcare advice, not necessarily from doctors but from, let’s say, elders within their family society, local community places of worship.’*

(GP1)

These alternative sources of information, and lack of trust in UK-based authorities, were considered to have created particular confusion and mistrust among migrant communities during the pandemic. Several migrants and clinicians stated that trust of a specific practice or individual is essential, but harder to build in the absence of face-to-face interactions:

*‘I find that if you do spend a bit more time with people right at the beginning … that then makes future consultations much more straightforward. Because you’ve done the relationship-building bit of that, and I think that’s much harder to do via telephone.’*

(GP22)

Several HCAs and PNs commented on the possible politicisation of doctors and were concerned around the link between health care and immigration status, particularly with regards to undocumented migrants and charging for care:

*‘People are wondering if you’re wanting to shop them in to the immigration police, or whether you think they haven’t been here long enough to qualify for NHS care ... that was a big barrier for some of our most vulnerable undocumented migrant patients in seeking care here because they were sure, and quite correctly, that their whereabouts are being shopped to the immigration authorities.’*

(GP16)

#### Indirect discrimination

Several migrants suggested that pandemic-related changes in primary care have utilised a ‘one-sizefits-all’ approach, but flexibility is essential to ensure equitable access. Practices’ approaches to digitalisation often failed to consider the needs of marginalised groups, and took a rigid approach to not seeing patients face-to-face, even if communication challenges would significantly affect consultation quality:

*‘They should not use just one way of contact which is like via the phone … please find some way to help. Rather than just putting the blame on that patient … Not everybody has the same opportunity or access.’*

(Migrant 4)

*‘I think that would be better if they would have a little bit GPs open so we could talk with them, because normal GPs have access to the interpreters. But it was completely shut down* [during the pandemic] *which is also a terrible thing to do.’*

(Migrant 1)

Both migrants and primary care professionals recognised that the physical closure of surgeries during the pandemic led to indirect discrimination because migrants had lost practical support from receptionists, and may no longer receive signposting, screening services, and new-patient health checks:

*‘So, although our registration seems easy, in COVID I expect it’s really difficult for people, because they can’t just walk in and get forms and do it in the waiting room. At least our receptionists speak a mixture of languages. They could help people fill in the forms.’*

(GP18)

### Risk factors for COVID-19 and concerns about COVID-19 vaccination roll-out

Primary care professionals and migrants alike reported concerns that preexisting distrust of vaccinations and the NHS, alongside low health literacy and widespread misinformation, were likely to negatively affect uptake of a COVID-19 vaccine in some migrants:

*‘We’re going to have major issues, because I think there’s, as I said before, there’s a huge distrust around the government.’*

(HCA6)

*‘Especially with the migrant patients, they’re not very accepting of other vaccinations ... it would be very hard ... The ones that probably are coming ... they have a flu, I think we might be able to convince them.’*

(PN3)

A number of migrants reported accessing contradictory information from different information sources, confusion, or indecision about whether to have the COVID-19 vaccine. A variety of information sources were described to support decision making, including advice from peers, social media, religious leaders, or information from country of origin:

*‘Or the country that you have left, you are still very closely linked to and therefore in that country they may have very strong views about things. You might still be swayed by those views rather than what’s in the mainstream of the countries that you’ve moved to. I see that as a problem.’*

(PN2)

*‘Actually the social media only, it was help for us* [in deciding about the vaccine] *, I was supported, yes, and everything, especially some of our community,* [home country] *community, in the UK, and they shared the information, which area is locked down ... ’*

(Migrant 11)

*‘Our church leaders, they’re all saying to us not to be vaccinated. And, to be honest, I have no right correct answer, I’m confused. I’m not 100% sure that this is actually connected with demonic people who are trying, how can I say, to put their power on people.‘*

(Migrant 15)

A range of specific beliefs around COVID-19 were reported, and it was perceived that these might contribute to reduced vaccine uptake in some migrant groups. These included the idea that COVID-19 is a hoax, a *‘European infection’* (PN13) or condition that is less likely to affect BAME groups, and a reliance on home remedies to protect against the effects of COVID-19:

*‘Some of the people, culturally, they don’t believe that such a virus exists. They think that it’s 5G or something else.’*

(Migrant 9)

*‘In a lot of ethnic countries, COVID hasn’t had the same impact as it has especially within Western Europe and America, and I think it’s being seen as very much a European infection. It doesn’t impact on BAME communities as much as they say ... There’s racial connotations to it as well, but any vaccine that they bought will be similar to the AIDS infection in Africa and people bringing infections to new countries.’*

(PN13)

*‘Most of the African-descended origin genes are resistant to the COVID. I don’t know if you’ve heard about that. Or maybe just something that they are talking about on YouTube.’*

(Migrant 5)

*‘You just give one teaspoon honey with seven flaxseeds every morning just give them, just to protect from viral.‘*

(Migrant 3)

A range of beliefs were also reported about the COVID-19 vaccines, ranging from general concerns that the vaccine will not work, will not be safe, or could result in contracting COVID-19, through to concerns about a conspiracy relating to the vaccine, and its ability to control or microchip people:

*‘They fear catching the vaccine and they would fear that it is a mass immunisation programme, that there could be lots of people around.’*

(PN11)

*‘There is fears about vaccine safety, people are scared of it, is it safe? Isn’t it safe?’*

(Migrant 14)

*‘He said, a doctor? So glad, you can tell me. Is it true, the vaccine for this virus, that it’s going to be microchip in it and track me around?’*

(GP16)

A number of migrants also reported concerns about discrimination or unequal approaches to research and vaccine distribution across ethnic groups. These included beliefs that their communities would not be represented in clinical trials, and concerns that they would be used as guinea pigs or the last community to receive it:

*‘The main problem is that we not having the same community participating too much in the trials.’*

(Migrant 5)

*‘If I go there* [for a vaccine] *they might be using me as a guinea pig or I don’t know. They might be using me for their own things. I don’t trust them.’*

(Migrant 9)

*‘We have that feeling we’d be the last to have the vaccination. Yes, we have that’*

(Migrant 16)

### Opportunities and solutions to inform the public-health response

Primary care professionals reported innovative solutions that could strengthen engagement with marginalised groups such as migrants, and have even led to new ideas to inform service delivery beyond the pandemic. These are outlined in Table 4 and included targeted, translated health advice and new community outreach approaches to faith and community leaders to access their communication networks and tackle misinformation.

**Table 4.** Key barriers and identified solutions

**Domain**	**Key finding**	**Solutions for primary care**
**New and exacerbated barriers to health care**	Increased use of technology	Use multiple modes of translated communications in combination, for example, text, emails, and leaflets Facilitation of virtual group consultations for patients with a specific condition, with a translator Option for the patient to send a written electronic message requesting a consultation and interpreter, and for GP to contact to follow-up within 24 hours (for example, by using eConsult)
	Socioeconomic challenges	Use social prescribers and broad multidisciplinary working, including the third sector Targeted access slots, for example, saved on-the-day appointments for marginalised patients or shift workers Service information or location sharing, for example, vouchers for food banks and virtual leaflets linking migrants to third-sector organisations addressing areas of need
	Communication barriers	Funding of translators and migrant community volunteers and champions Working with local media and communication hubs to deliver COVID-19 health information
	Trust, authority, and information	Relationship building from registration, for example, a new-migrant patient health check Targeted information through local leaders, social media, and traditional means, such as posters and flyers, about the NHS, links between health and immigration services including information-sharing about immigration status, COVID-19, and pandemic-related system changes
	‘One-size-fits-all’ approach	Be flexible in bringing patients in, requesting patients attend the practice for a face-to-face consultation if no other method of communication is possible or effective Widespread diversity training and ‘migrant-friendly’ practices, for example, using the Safe Surgeries scheme^a^

**Key risk factors for health and COVID-19 vaccine roll-out**	Challenges identifying migrants	Effective coding of country of origin, language, and ethnicity to enable identification when patients register with a practice
	Migrants have more risk factors for COVID-19	Integrated ‘one-stop shop’ screening and new-migrant patient health checks
	Challenges decision making about the COVID-19 vaccine	Outreach to local leaders to support community decision making around the vaccine
	Beliefs about COVID-19 and the COVID-19 vaccine	Seek to understand the perspective of local migrant communities, for example, through patient champions and patient participation groups, and distribute accurate, translated information

**Opportunities and solutions to improve access to primary care**	New technology for service delivery	New models, such as virtual group consultations, use of tailored translated texts, and text templates (for example, using accuRx to send translated screening invitations) to encourage access from marginalised groups
	Codesign of new models	Encourage involvement throughout service design, including proportionate representation in patient participation groups, trials, and information campaigns
	Specialist input across a group of practices	Clinical commissioning group or primary care network funding of specialist support services, for example, through a local enhanced service

a *The Safe Surgeries initiative is run by Doctors of the World. A Safe Surgery is any GP practice that commits to taking steps to tackle the barriers faced by many migrants in accessing health care.*

New specialist services were mentioned, including specifically funded services across multiple practices to coordinate interpreting and volunteer services. There was consensus that clear, concise, and language-specific written and non-written resources needed to be developed — ideally by central bodies such as the UK Government or Public Health England — for local distribution to facilitate COVID-19 vaccine uptake in migrant groups. Participants noted that clinical commissioning groups, GP practices, and pharmacies must proactively reach out to migrant communities and their institutions to codesign solutions:

*‘There could be some great accuRx* [text-messaging] *templates for new migrant patients … “Have you recently arrived in the UK? Would you like to get some health screening? ”’*

(GP16)

*‘Who are the faith leaders of those communities, who runs them, and how are they communicating at the moment? For example, if you look at people who are Muslim, they’re not going to pray together … the information they’re getting must be coming from their faith leaders. They must be having the call to prayer; there must be a communication network to do that.’*

(GP8)

*‘And I don’t mean just written information* [about the COVID-19 vaccines] *, there needs to be more than that because, obviously, a lot of these people don’t actually read the language that they speak. So, it has to be more sort of interpreted and contextualised through that to sort of, perhaps, work with the population, maybe with the religious leaders and so on to get people involved.’*

(GP11)

## DISCUSSION

### Summary

This study reports the perspectives of recently-arrived migrants and primary care professionals on the impact of COVID-19 on UK migrant communities, their access to health care, and their views around the roll-out of COVID-19 vaccines. It was found that digitalisation had exacerbated existing inequalities regarding access for some migrant groups through a lack of access to, or knowledge of, technology; concerns about language barriers, difficulties building trust, and the risk of missing safeguarding cues in virtual consultations were also expressed. The physical closure of some surgeries was reported to have led to challenges in migrants registering with, and accessing, primary care.

Communication barriers, feeling left behind in receiving support and health interventions in comparison to the general population, and a lack of access to information were issues widely raised by migrants. Additionally, they reported views of COVID-19 and COVID-19 vaccinations that ranged from acceptance to misinformation, often originating from social media or word of mouth. Some migrants experienced increased risk factors to their health and severe illness from COVID-19, partially resulting from their economic and social situations.

Primary care professionals reported that pandemic-related changes to healthcare delivery may become permanent, and expressed how issues could be tackled through innovations in service delivery, such as translated health advice using text templates and YouTube.

### Strengths and limitations

This study has generated valuable insights into the experiences of migrants during the COVID-19 pandemic, and has direct and immediate relevance to the ongoing public-health response. The scale of this study — the use of multiple phases building iteratively on one another in data collection and analysis, and engagement of diverse voices (including migrants and a range of different primary care professionals) — enhance its validity. The similarities in perspectives between migrants and professionals were striking, providing corroboration across groups for the findings.

Migrants are a diverse group, and the present study focused only on a relatively small sample of recently-arrived migrants. Therefore, next steps would be to engage migrants from all dominant nationality groups in the UK and different types of migrants (for example, labour, undocumented, and migrants who have been settled in the UK for a longer time) to explore the culture-specific impacts of COVID-19 and age-related differences.

Although participants were recruited from many different counties across England, the structure and experience of primary care differ in the devolved nations of the UK; this may limit the generalisability of the findings. In addition, the key researchers leading the interviewers were universally female, highly-educated, and White. Thus, the researchers’ ethnicities, social background, and professional training may have influenced responses, particularly of migrants, through perceived power differentials and social desirability bias.

The findings reported here may be of relevance for other marginalised groups who may lack digital literacy or access to technology, or may lack access to official sources of information, such as those who are homeless, Gypsy, Roma and traveller communities, and remote communities in the UK, as highlighted by recent rapid evidence reviews by the Royal College of General Practitioner’s Health Inequalities Forum.^18^

### Comparison with existing literature

Despite a growing body of research exploring the impact of COVID-19 on Black, Asian, and minority ethnic groups, the specific impact on migrant groups had not been explored in depth. Migrants may have unique risk factors and vulnerabilities to COVID-19^6^ and face barriers to health care and poor health outcomes in the UK and Europe,^3^ which the authors found were exacerbated during the pandemic due to digitalisation; however, digitalisation may negatively impact a range of marginalised groups in similar ways. A recent study of the impact of remote consulting in the UK across 21 practice populations similarly reported concerns by GPs that non-verbal cues are more important in migrant and other marginalised groups, and that *‘SMS, e-consultations, and video would increase access for those with IT skills, and enforce already existing health inequities’*.^9^ Doctors of the World UK concurs, reporting that individuals from a range of excluded groups lack access and skills to use technology, and face an inability to pay for access to broadband or mobile data; it also reports that migrants lacked access to key COVID-19 public-health messages in their own language.^12^ This was a key theme in the study presented here, which, combined with low health literacy, leaves these groups vulnerable to misinformation. This demonstrates the need for linguistically and culturally appropriate health information; the Swedish ‘corona lines’ — telephone services in various languages offering advice and COVID-19 triaging — are one example of good practice.^19^

Other studies conducted before the pandemic took hold are conflicting, suggesting there is a digital divide in resources needed to participate in remote consultations,^7^ and that minority groups, such as migrants, are more likely to miss virtual appointments,^20^ but can also benefit more than majority groups if interventions are specifically designed with their needs and capabilities in mind.^21^ Leite *et al* have highlighted that if barriers such as access and digital literacy can be overcome, digitalisation provides a crucial way to ease the impact of the pandemic.^22^ This concurs with the study presented here, which found that where migrants could access and use technology, translated texts and YouTube could provide health information and support access beyond the pandemic.

The present findings support existing literature regarding the need for flexible and adapted policies in order to minimise disparities,^23^ interventions to address structural inequalities,^4^^,^^12^ and a need to ensure the availability of culturally and linguistically appropriate information about COVID-19 and a COVID-19 vaccine.^24^

The findings presented here provide insight into factors likely to impact on a COVID-19 vaccine roll-out in migrant communities, and concur with previous studies in that migrants may trust social networks over medical professionals,^25^ may be more likely than the general population to believe COVID-19 misinformation,^26^ and may mistrust findings of COVID-19 vaccine research.^24^ Specifically engaging diverse migrant groups in the UK and codesigning interventions to facilitate COVID-19 vaccine uptake, is therefore a crucial next step.

### Implications for research and practice

Practices should seek to ensure they can identify migrants, that they understand their needs through proactive engagement, and that they are providing language-specific advice about COVID-19 and changes in service provision during the pandemic through multiple modalities (for example, by text, email, letter, and posters in local community hubs). As COVID-19 vaccines are rolled out, the findings presented here provide critical information about how migrants’ needs might be met. This could include using patient participation groups and other local community groups to codesign delivery approaches, ensuring the availability of interpreters and translated vaccine information that is culturally appropriate, integrating ‘migrant ambassadors’ into vaccine centres, and developing information-sharing campaigns.

Further research should compare and evaluate different virtual consultation approaches in other marginalised groups, as well as the success of codesigned service delivery models in improving access to health care and strengthening COVID-19 vaccine uptake.

Increased digitalisation of primary care delivery may become permanent. This study demonstrates that migrant groups, many of whom already face barriers to primary care, are at risk of digital exclusion and may need targeted additional support to access services — a finding that may be relevant for other marginalised groups. Migrants may also be at increased risk of misinformation about COVID-19 and face barriers to vaccination; participants emphasised the need to reach out to local community groups and to provide clear, concise, and language-specific written and non-written resources to facilitate COVID-19 vaccine uptake. As primary care networks adapt in the face of the COVID-19 pandemic, these findings provide critical information on specific strategies that may help support migrant groups to access primary care and overcome misinformation about COVID-19 and the COVID-19 vaccines.
